# Factors Associated With Severe Fever With Thrombocytopenia Syndrome in Endemic Areas of China

**DOI:** 10.3389/fpubh.2022.844220

**Published:** 2022-02-24

**Authors:** Xiaolin Jiang, Yiguan Wang, Xiaomei Zhang, Bo Pang, Mingxiao Yao, Xueying Tian, Shaowei Sang

**Affiliations:** ^1^Shandong Provincial Center for Disease Control and Prevention, Jinan, China; ^2^Shandong Key Laboratory of Infectious Diseases, Jinan, China; ^3^Ashworth Laboratories, Institute of Evolutionary Biology, University of Edinburgh, Edinburgh, United Kingdom; ^4^Clinical Epidemiology Unit, Qilu Hospital of Shandong University, Jinan, China; ^5^Clinical Research Center of Shandong University, Qilu Hospital, Cheeloo College of Medicine, Shandong University, Jinan, China

**Keywords:** severe fever with thrombocytopenia syndrome, climatic factors, environmental factors, nonlinear, socioeconomic factors

## Abstract

**Objective:**

To explore the influence of climatic, environmental and socioeconomic factors on SFTS occurrence in Shandong Province, China.

**Methods:**

We used generalized additive model to estimate the association between SFTS cases and climatic factors, environmental factors and socioeconomic factors, including annual average temperature, precipitation, land cover, normalized difference vegetation index, altitude, population density, meat production, milk production, and gross domestic product (GDP).

**Results:**

There were a total of 4,830 cases reported in 100 (70.9%) counties and districts in Shandong Province from 2010 to 2020. The results showed that the annual average temperature, precipitation, forest and grassland coverage rate, altitude and meat production (square root transform) had a reversed “V” relationship with SFTS occurrence, with the inflection points around 12.5–13.0°C in temperature, around 650 mm in precipitation, around 0.3 in forest and grassland coverage rate, around 300 m in altitude, and around 200–300 tons in meat production (square root transform), respectively. SFTS occurrence had a “V” relationship with milk production (square root transform) and GDP (square root transform), with the inflection points around 100–200 tons in milk production (square root transform), and around 150,000–200,000 yuan in GDP (square root transform), respectively.

**Conclusions:**

Climatic, environmental, and socioeconomic factors contributed to the heterogeneous distribution of SFTS in Shandong Province, and the influence of these factors on SFTS occurrence was nonlinear.

## Introduction

Severe fever with thrombocytopenia syndrome (SFTS) is an emerging infectious disease, which was firstly reported in rural areas of Hubei Province and Henan Province, China in 2009 (1). The pathogen for SFTS was identified as a novel *phlebovirus* of *Bunyaviridae* family, and designated as SFTS *bunyavirus* (SFTSV) by Yu's team (1). Currently, SFTSV has been assigned to genus *Bandavirus* in family *Phenuiviridae* of *Bunyavirales* by International Committee on Taxonomy of Viruses (ICTV). After the identification of SFTSV, SFTS cases have been subsequently reported in Japan (2), Korea (3), and Vietnam (4). Given the potential threat to public health, SFTS was listed as one of the 9 emerging diseases prioritized for research and development by the World Health Organization (WHO) in 2017 (5).

SFTS has been listed as a notifiable disease in China since 2010. The number of SFTS cases have been continuously growing with an expansion of geographical distribution over the past decade in China. The laboratory confirmed cases increased from 461 in 2011 to 1,209 in 2018 (6). There were 11 provinces reporting SFTS cases in China in 2012 (7), but the number increased to 23 in 2016 (8) and 25 in 2018 (9).

Several clusters of SFTS cases have been reported in China, suggesting a human-to-human transmission of the disease (10, 11), but SFTS is mainly considered a vector-borne infectious disease and *Haemaphysalis longicornis* is thought to be the primary vector of SFTSV (12). *Haemaphysalis longicornis* has a wide range of hosts, including domesticated and wild animals. There is evidence indicating that the domesticated animals, rodents and shrews have been widely infected by SFTSV in the endemic areas and they may act as amplifying hosts of SFTSV (13). Therefore, SFTSV is recognized to circulate in an enzootic tick-host-tick cycle.

SFTS is a life-threatening disease and the average fatality rate was estimated at 10.5% (95% CI: 9.8–11.2%) (9). Currently, there is no available specific treatment or vaccine for SFTS. Prevention may be the best strategy to control the spread of the disease and reduce the disease burden. Thus, the knowledge of risk factors associated with SFTS occurrence is of great public health significance.

The previous study showed that there were four geographical clusters of SFTS cases in China, with two of them in Shandong Province and one of the clusters had the highest average annual incidence in China (9). A study conducted in two counties of Shandong Province showed that SFTSV-specific antibodies were detected in 69.5% of sheep, 60.5% of cattle, 47.4% of chickens, 37.9% of dogs, and 3.1% of pigs, and the sequences of SFTSVs from these domesticated animals were highly similar (>95% homology) to human isolates from the regions (14). In this study, we choose the endemic area, Shandong Province as the study area to explore the influence of climatic factors, environmental factors, and socioeconomic factors on SFTS occurrence.

## Materials and Methods

### Study Area

The National Census Bureau showed that the number of residents in Shandong Province was over 0.1 billion in 2020, which makes Shandong the second populated province in China. Shandong is a coastal province located in the east of China (34°22.9' - 38°24.01' N, 114°47.5' - 122°42.3' E) with a temperate monsoon climate. The annual average temperature is 12–16°C. The annual average precipitation is 450–700 mm around. The area of Shandong Province is 158,000 km^2^, with mountain and hilly areas accounting for approximately 15% and woodland areas for ~16%. County or district is the study unit. Shandong Province is administratively divided into 141 counties or districts.

### Data Collection

Data on confirmed SFTS cases from January 2010 to December 2020 in Shandong Province were downloaded from China Notifiable Disease Surveillance System. Each case was confirmed by laboratory or clinical diagnosis. Information of SFTS cases included age, gender, residential address, type of diagnosis, and date of onset. The number of confirmed SFTS cases from January 2010 to December 2020 was aggregated at the county or district level.

Climatic data including 1 km monthly mean temperature and 1 km monthly precipitation dataset for China from 2010 to 2020 were downloaded from the National Earth System Science Data Center, National Science and Technology Infrastructure of China (http://www.geodata.cn). We extracted and averaged monthly mean temperature and precipitation within each county or district in Shandong Province from 2010 to 2020.

Environmental data on 1 km altitude, 1 km normalized difference vegetation index (NDVI) for China from 2010 to 2019, 1km land cover types for Shandong Province in 2020 were downloaded from Resource and Environment Science and Data Center (https://www.resdc.cn/). Among various land cover types, the forest and grassland coverage rate was selected for the study, considering the influence on the SFTS occurrence.

Socioeconomic data including 1 km gross domestic product (GDP) for China in 2015 and 1 km population size for China in 2015 were downloaded from Resource and Environment Science and Data Center (https://www.resdc.cn/), and data on meat production and milk production of each county or district in 2015 were downloaded from Shandong Statistic Year Book.

### Statistical Analyses

The generalized additive model (GAM) was used to explore the linear or nonlinear relationship between SFTS occurrence and the climatic, environmental and socioeconomic variables. Because SFTS cases were over-dispersed, we used *quasi-*poisson distribution to allow for over-dispersion data. Spearman correlation analysis showed that two variables, altitude and forest and grassland coverage rate, were highly correlated, with a correlation coefficient of 0.8. Therefore, the two variables were separated into two models with other explanatory variables to build the multivariate GAM models.


$$\begin{array}{l} \text{log}\left(u_{i}\right)=b_{0}+s\left(x_{i1}\right)+s\left(x_{i2}\right)+s\left(x_{i3}\right)+\ldots +s\left(x_{ij}\right) \end{array}$$

*u*_*i*_ represents expected SFTS cases in the *i*th county or district. *x*_*ij*_ represents annual average temperature, precipitation, NDVI, meat production (square root transformation), milk production (square root transformation), GDP (square root transformation), population size, and forest and grassland coverage rate in model 1; and annual average temperature, precipitation, NDVI, meat production (square root transformation), milk production (square root transformation), GDP (square root transformation), population size, and altitude in model 2. s() represents the function used in the definition of smooth terms within GAM model formulae. In this study, a thin plate regression spline was used as the penalized smoothing basis. The smoothing parameters were estimated using the restricted maximum likelihood (REML) method. To avoid unstable estimates, the concurvity between the smooth terms was checked (15). The sensitivity of the trend was tested by replacing the thin plate regression spline with a cubic regression spline as the smoothing basis. To quantify the presence of spatial autocorrelation in the residuals from the models we computed Moran's I statistic (16) and conducted a permutation test to assess its significance. All the statistical analyses were performed by R 4.1.0 (28).

### Ethics Statement

Ethical approval for the study was obtained from Qilu Hospital of Shandong University Ethical Committee (No. KYLL-2021(KS)-056).

## Results

**Figure 1:**
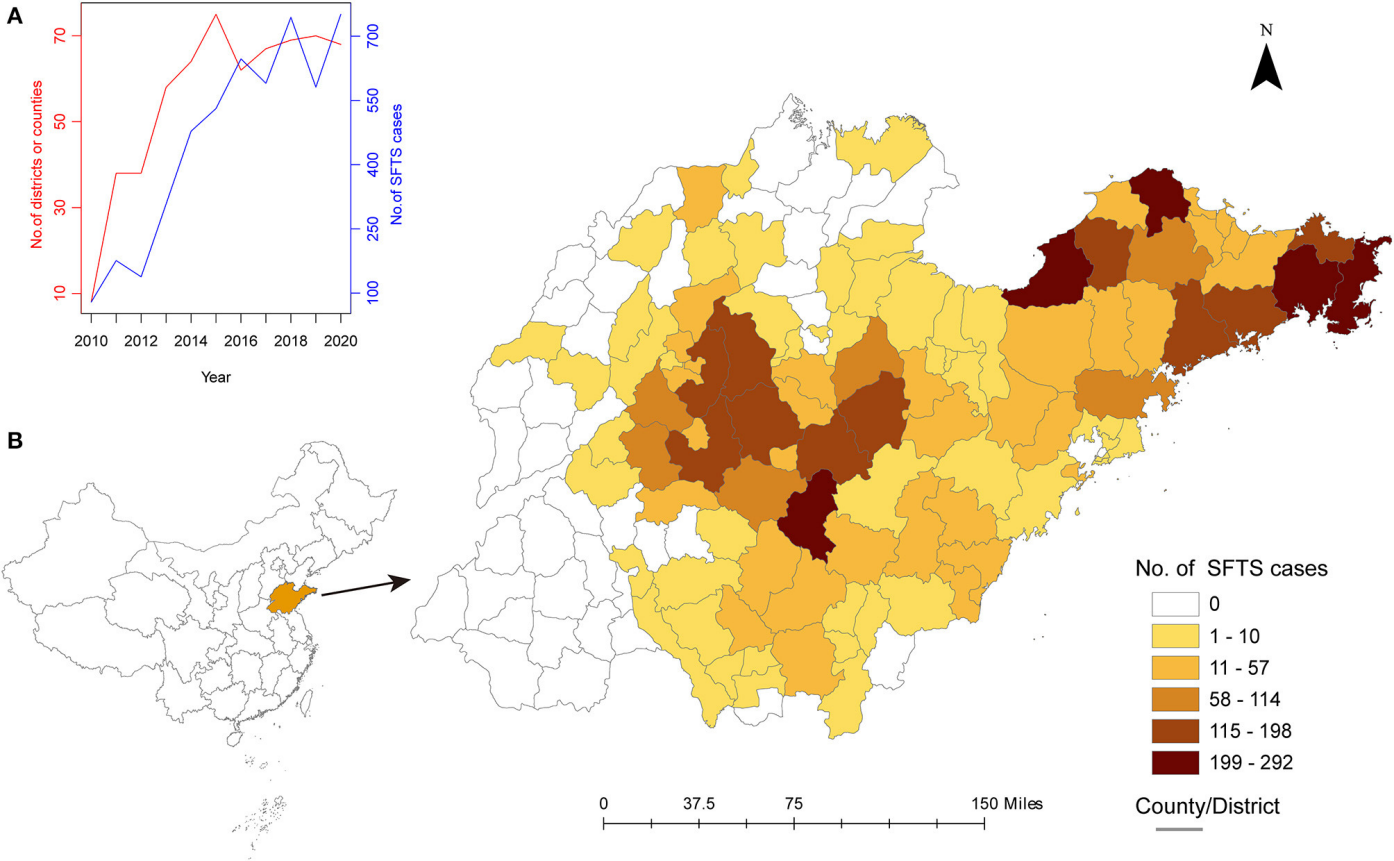
The epidemiological characteristics of SFTS cases in Shandong Province from 2010 to 2020. **(A)**, the trend of the number of SFTS cases and number of locations reporting SFTS cases. **(B)**, the spatial distribution of SFTS cases in Shandong Province.

There were a total of 4,830 SFTS cases reported from 100 (70.9%) counties or districts in Shandong Province from 2010 to 2020. The annual number of SFTS cases increased from 79 in 2010 to 751 in 2020. The number of counties or districts reporting SFTS each year increased from 8 in 2010 to 68 in 2020, with the highest number of 75 occurring in 2015 (Figure 1A). The spatial distribution of SFTS cases was heterogeneous, with cases mainly clustered in the Shandong Peninsula and middle of the province while fewer cases in the west, southwest and north of the province (Figure 1B).

**Figure 2:**
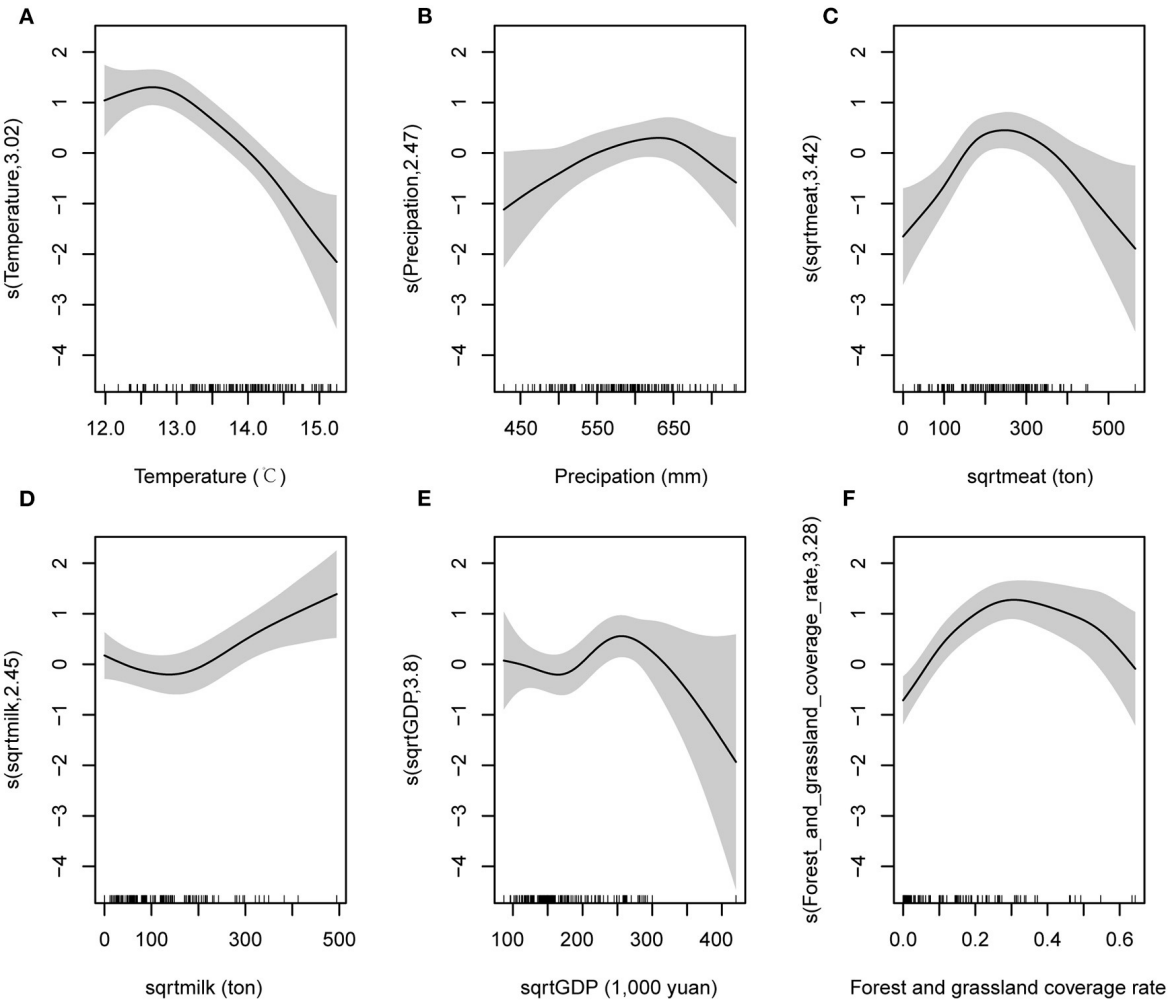
The factors that associated with SFTS occurrence and the relationships between these factors and SFTS occurrence, which were obtained from model 1. Variable sqrtmeat is meat production with square root transformation. Variable sqrtmilk is milk production with square root transformation. Variable sqrtGDP is GDP with square root transformation.

**Figure 3:**
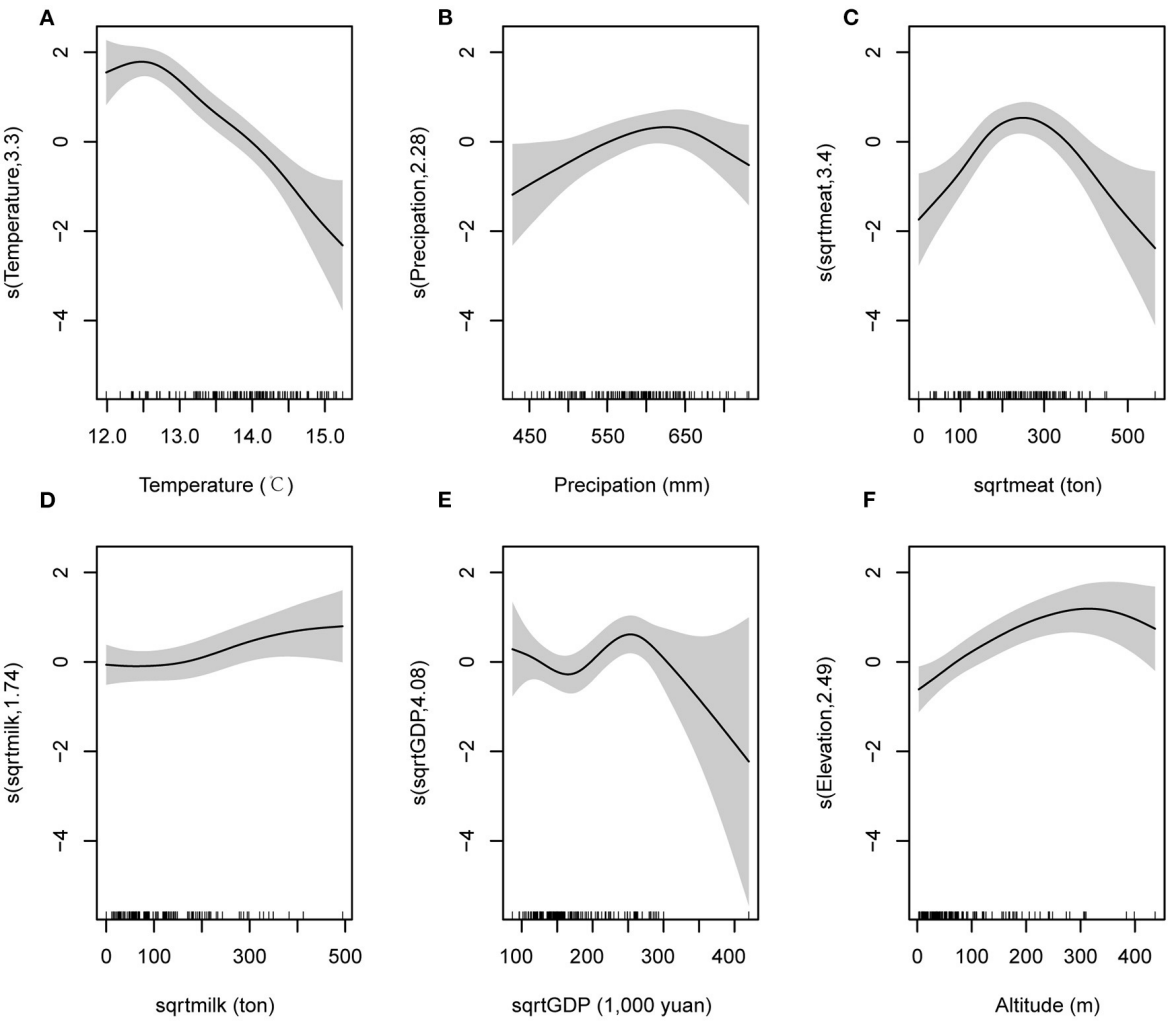
The factors associated with SFTS occurrence and the relationships between these factors and SFTS occurrence, which were obtained from model 2. Variable sqrtmeat is meat production with square root transformation. Variable sqrtmilk is milk production with square root transformation. Variable sqrtGDP is GDP with square root transformation.

We used GAM to investigate the relationships between SFTS risk and the annual average temperature, precipitation, meat production (square root transformation), milk production (square root transformation), GDP (square root transformation), forest and grassland coverage rate, NDVI, and population. The models showed that SFTS occurrence was associated with annual average temperature, precipitation, meat production (square root transformation), milk production (square root transformation), GDP (square root transformation), forest and grassland coverage rate, and altitude (Figures 2,3). Specificly, the model 1 showed that the relationships between SFTS occurrence and annual average temperature, precipitation, meat production (square root transformation), and forest and grassland coverage rate were reversed “V”-shaped. The risk of SFTS occurrence increased with the annual average temperature when it was below 12.5°C, but a dramatically decreased risk was observed when it was above 13°C (Figure 2A). The highest risk of SFTS occurrence was identified around 650 mm for the annual average precipitation (Figure 2B), around 200–300 tons for meat production (square root transformation, Figure 2C), and around 0.3 for forest and grassland coverage rate (Figure 2F). SFTS occurrence had a “V”-shape relationship with milk production (square root transformation), with the lowest risk identified around 100–200 tons for milk production (square root transformation) (Figure 2D). Within the range of 0–300,000 yuan, SFTS occurrence also had a “V”-shape relationship with GDP (square root transformation), with the lowest risk identified around 150,000–200,000 yuan for GDP (square root transformation) (Figure 2E).

In the second model (model 2) which was derived from model 1 by substituting the forest and grassland coverage rate with altitude, the estimated effects of annual average temperature, precipitation, meat production (square root transformation), milk production (square root transformation), and GDP (square root transformation) on SFTS occurrence were similar with those in model 1 (Figure 3). In addition, we observed a reversed “V”-shaped relationship between SFTS occurrence and the altitude. Within the range of 0–300 meters, the SFTS occurrence gradually increased with the altitude, and then gradually decreased when the altitude was higher than 300 meters (Figure 3F).

The sensitivity analysis was conducted by replacing the thin plate regression spline with a cubic regression spline as the smoothing basis in GAM models. The results showed that the associations and trends of annual average temperature, precipitation, meat production (square root transformation), milk production (square root transformation), GDP (square root transformation), forest and grassland coverage rate, and altitude with SFTS occurrence were stable (Supplementary Figures 1, 2).

The Moran's I tests of all the models had a *p*-value larger than 0.05, which suggested that the residuals didn't contain spatial autocorrelation. Concurvity analysis showed there was no concurvity in the data.

## Discussion

Although severe fever with thrombocytopenia syndrome (SFTS) was firstly reported in Hubei Province and Henan Province in the middle of China, the most affected province was Shandong where there were a significant increase of the cases and an expansion of the geographic regions for SFTS from 2010 to 2020. The spatial distribution of SFTS cases in Shandong Province is highly heterogeneous, with most cases clustered in the Shandong Peninsula and the middle of the province. SFTS is a tick-borne disease and we tried to explore the influence of climatic, environmental, and socioeconomic factors on SFTS distribution in this study. Our results show that annual average temperature, precipitation, altitude, land cover, meat production, milk production, and GDP had a nonlinear relationship with SFTS occurrence in Shandong Province.

Epidemiological studies have shown that SFTS occurrence had seasonal fluctuations, with the main epidemic seasons observed between May and July in China (8), between July and October in South Korea (17), and between May and August in Japan (18), which indicates that climatic factors play important roles in SFTS occurrence.

Several studies have investigated the association between SFTS occurrence and temperature and precipitation. Some of these studies assumed a linear relationship while the other studies explored the nonlinear relationship. A study conducted in Hubei Province, central China showed that SFTS occurrence was negatively linearly associated with monthly temperature, and had no significant association with monthly precipitation (19). A national-wide study in China from 2010 to 2018 showed that precipitation had a positive linear association with SFTS occurrence (9). A nonlinear relationship with monthly mean temperature and precipitation was observed in both a study conducted in Zhejiang Province from 2011 to 2018 (20) and a national-wide study from 2010 to 2013 (21). Using ecological niche modes, Sun et al. also found that SFTS occurrence had a nonlinear relationship with annual average temperature and precipitation (6), which is quite consistent with our results.

Temperature and precipitation can influence SFTS occurrence by directly altering the life cycle of ticks and their spatial distribution (22, 23). Within a favorable temperature range, higher temperatures can shorten tick development and growth periods, increase egg productivity and hatch-ratios (24). The increase of precipitation could create more breeding sites in shrub or forest areas and hence increase the population of the ticks. However, studies also showed that extreme temperatures could reduce ticks hatch-ratios, increase the mortality rates at molting or questing stages (25, 26). Excessive precipitation could flush out the tick breeding sites and destroy eggs and larvae. Therefore, temperature and precipitation are expected to exhibit a nonlinear influence on the life cycle of ticks and thus on the occurrence of SFTS.

A growing body of evidence showed that the SFTS occurrence was associated with altitude. Miao et al. found that the association was linear and highly positive (Hazard ratio 2.04, 95% CI: 1.87–2.24) (9). Several other studies found that the risk of SFTS occurrence had a reversed “V” relationship with altitude (6, 23), which was consistent with the observations in our study. The possible reasons may include that (1) the ticks and hosts mainly distribute in the areas with relatively lower altitude; (2) the higher the altitude is, the fewer outdoor activities humans would have and thus less likely to be exposed to ticks. In our study, altitude and forest and grassland coverage rate were highly correlated. The risk of SFTS occurrence also showed reversed “V” relationship with forest and grassland coverage rate. Several studies have investigated the role of land cover on SFTS occurrence. Miao et al. found that the coverages of woodland, rainfed cropland and shrubland were highly associated with the spatial distribution of SFTS occurrence (9). Liu et al. indicated that forest coverage was an important factor in determining the spatial distribution of SFTS occurrence (21). A possible explanation is that forest areas, an attractive landscape for humans to visit or work, have considerable populations of reservoir hosts and high densities of ticks (26), which increases the risk of direct contact with ticks.

The association between livestock and SFTS occurrence is controversial among studies. Liu et al. indicated that cattle density was associated with SFTS occurrence but goat density did not (21). Sun et al. showed that goat density, cattle density, poultry density and swine density had no significant association with SFTS occurrence but sheep density had (6). Miao et al. found that livestock density could be used to predict the spatial distribution of *Haemaphysalis longicornis* and SFTS cases (23). In our study, we found that meat production and milk production were nonlinearly associated with SFTS occurrence. The hosts of ticks are wide and cover a variety of domesticated animals, and the infection rates of SFTSV among these hosts in different areas are also heterogeneous, which may explain the contradictory results from different studies.

Our study used a high-resolution dataset to explore the associated factors with SFTS occurrence. However, there are still some limitations. The data on SFTS cases were obtained from China Notifiable Disease Surveillance System, which is passive surveillance. Thus, the SFTS cases might be underreported in the system (27), particularly in the less developed areas due to the insufficient virologic diagnostic and reporting capacity. In addition, the increase of the SFTS cases and the locations reporting SFTS cases could partly attribute to the increase of the diagnostic capacity from 2010 to 2020. Besides, SFTS is a tick-borne disease, but we cannot obtain the data on the distribution of vertebrate hosts of tick. In this study, we intended to use meat production and milk production as a proxy to represent the livestock information, which may accurately reflect the wide range of hosts.

## Conclusions

SFTS is endemic in Shandong Province with an increasing number of SFTS cases and an expansion of its distribution. Climate, environmental, and social and economic development may contribute to the distribution of SFTS occurrence. Here, we studied the potential risk factors of annual average temperature, precipitation, meat production (square root transformation), milk production (square root transformation), GDP (square root transformation), altitude, and forest and grassland coverage rate. The relationships between these factors and SFTS occurrence are nonlinear. The study will help to better understand the effect of these factors on SFTS and may have policy implications for disease prevention and control.

## Data Availability Statement

The original contributions presented in the study are included in the article/Supplementary Materials, further inquiries can be directed to the corresponding author/s.

## Author Contributions

XJ: investigation, data curation, data analysis, writing-original draft, and writing-review and editing. YW: data analysis, writing-original draft, and writing-review and editing. XZ, BP, MY, and XT: investigation and writing-review and editing. SS: surveillance, data analysis, writing-original draft, writing-review and editing, and funding. All authors contributed to the article and approved the submitted version.

## Funding

This study was supported by Shandong Natural Science Foundation (ZR2021MH242, ZR2014HP030) and Shandong medical and health science and technology development plan (2018WS306). The funders had no role in the study design, data collection and analysis, decision to publish, or preparation of the manuscript.

## Conflict of Interest

The authors declare that the research was conducted in the absence of any commercial or financial relationships that could be construed as a potential conflict of interest.

## Publisher's Note

All claims expressed in this article are solely those of the authors and do not necessarily represent those of their affiliated organizations, or those of the publisher, the editors and the reviewers. Any product that may be evaluated in this article, or claim that may be made by its manufacturer, is not guaranteed or endorsed by the publisher.

## Supplementary Material

Supplementary Figure 1 The result of sensitivity analysis from model 1. Variable sqrtmeat is meat production with square root transformation. Variable sqrtmilk is milk production with square root transformation. Variable sqrtGDP is GDP with square root transformation.

Supplementary Figure 2 The result of sensitivity analysis from model 2. Variable sqrtmeat is meat production with square root transformation. Variable sqrtmilk is milk production with square root transformation. Variable sqrtGDP is GDP with square root transformation.
