# Supplementary figures and images for: Factors Associated With Severe Fever With Thrombocytopenia Syndrome in Endemic Areas of China
Source: Front Public Health. 2022 Feb 24;10:844220. doi: 10.3389/fpubh.2022.844220 (PMC8907623; doi:10.3389/fpubh.2022.844220)

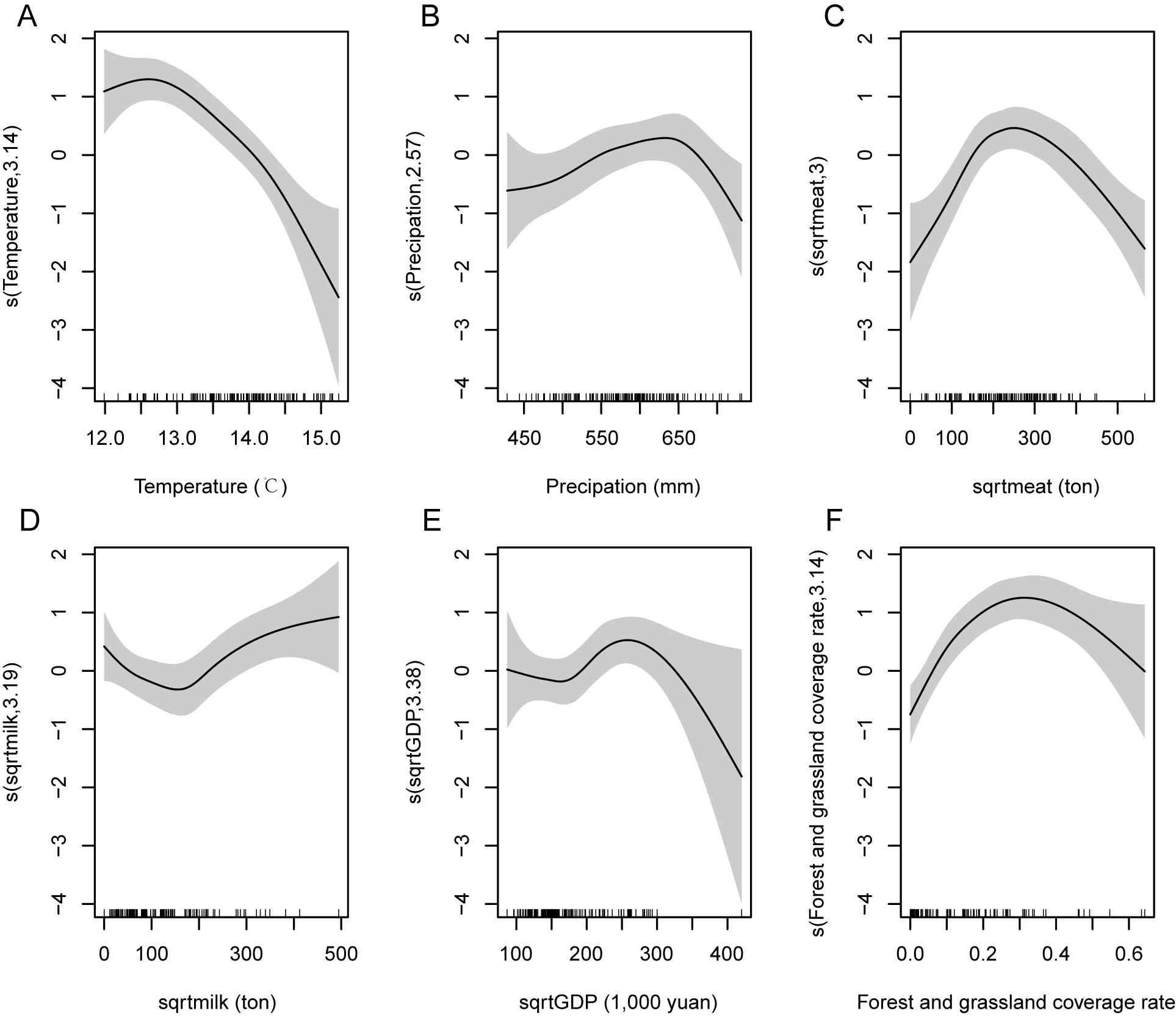

Supplement: Supplementary Figure 1 — The result of sensitivity analysis from model 1. Variable sqrtmeat is meat production with square root transformation. Variable sqrtmilk is milk production with square root transformation. Variable sqrtGDP is GDP with square root transformation. [file Image_1.TIF]

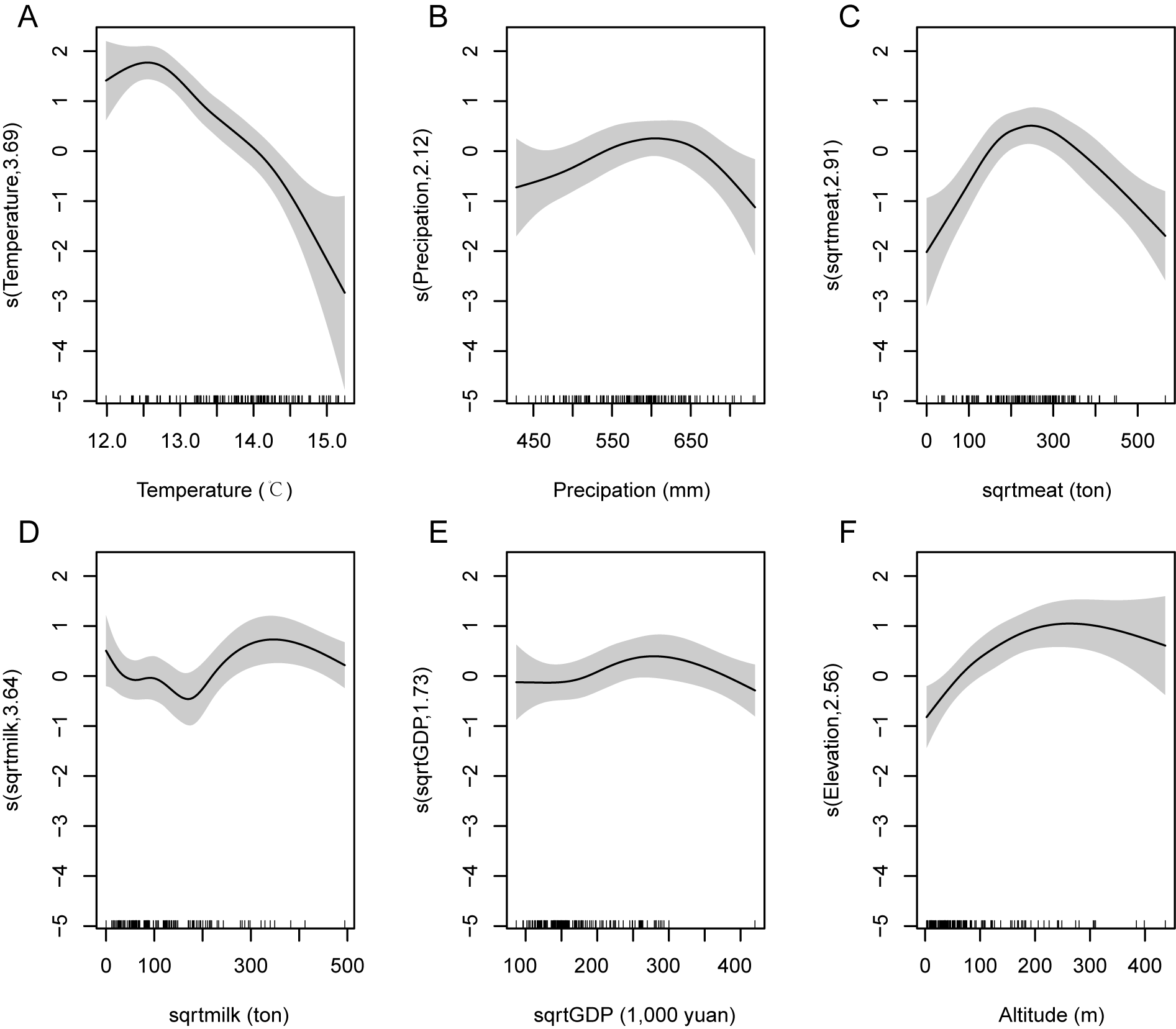

Supplement: Supplementary Figure 2 — The result of sensitivity analysis from model 2. Variable sqrtmeat is meat production with square root transformation. Variable sqrtmilk is milk production with square root transformation. Variable sqrtGDP is GDP with square root transformation. [file Image_2.TIF]
